# Spatial Epidemiologic Trends and Hotspots of Leishmaniasis, Sri Lanka, 2001–2018

**DOI:** 10.3201/eid2601.190971

**Published:** 2020-01

**Authors:** Nadira D. Karunaweera, Samitha Ginige, Sanath Senanayake, Hermali Silva, Nuwani Manamperi, Nilakshi Samaranayake, Yamuna Siriwardana, Deepa Gamage, Upul Senerath, Guofa Zhou

**Affiliations:** University of Colombo, Colombo, Sri Lanka (N.D. Karunaweera, S. Senanayake, H. Silva, N. Samaranayake, Y. Siriwardana, U. Senerath);; Ministry of Health, Colombo (S. Ginige, D. Gamage);; University of Kelaniya, Ragama, Western Sri Lanka (N. Manamperi);; University of California Irvine, Irvine, California, USA (G. Zhou)

**Keywords:** *Leishmania donovani*, epidemiology, protozoa, parasites, cutaneous leishmaniasis, skin lesions, dermatological pathologies, infectious diseases, vector-borne infections, Asia, Indian subcontinent, Sri Lanka, leishmaniasis

## Abstract

Leishmaniasis, a neglected tropical disease, is on the decline in South Asia. However, cases of cutaneous leishmaniasis have risen in Sri Lanka since 2001, and the lack of in-depth research on its epidemiologic characteristics hampers control efforts. We analyzed data collected from patients with cutaneous leishmaniasis in Sri Lanka during 2001–2018 to study temporal and geographic trends and identify and monitor disease hotspots. We noted a progression in case rates, including a sharp rise in 2018, showing temporal expansion of disease-prevalent areas and 2 persistent hotspots. The northern hotspot shifted and shrank over time, but the southern hotspot progressively expanded and remained spatially static. In addition, we noted regional incidence differences for age and sex. We provide evidence of temporally progressive and spatially expanding incidence of leishmaniasis in Sri Lanka with distinct geographic patterns and disease hotspots, signaling an urgent need for effective disease control interventions.

Leishmaniases are diseases caused by *Leishmania* spp. parasites transmitted through the bites of infected female phlebotomine sand flies. A neglected tropical disease that mainly affects the tropics and subtropics, leishmaniasis has 3 forms: cutaneous, visceral, and mucocutaneous (*1*). Cutaneous leishmaniasis (CL) is the most common form, causing skin lesions that can leave scars and cause lifelong disability (*1*). Visceral leishmaniasis (VL) is the most serious form and has a case-fatality rate >95% in untreated cases; globally, 50,000–90,000 new cases and 20,000–40,000 deaths occur annually, making VL one of the largest killers among neglected tropical diseases (*1**–**3*). Approximately 0.7–1 million new CL cases and a few thousand mucocutaneous leishmaniasis cases occur worldwide each year (*1**–**3*). 

South Asia has the highest incidence of VL; India, Nepal, and Bangladesh are predominantly affected. Leishmaniasis in this region is caused by *Leishmania* *donovani* transmitted by *Phlebotomus argentipes* sand flies (*2**–**4*). Driven by the goal to eliminate VL in South Asia by 2020, the 3 countries once highly endemic for VL have made remarkable progress, bringing down reported cases from 50,898 in 2007 to 6,174 in 2017; Nepal had an 84% case reduction, India an 87% reduction, and Bangladesh a 96% reduction (*2*,*4*). Such efforts have contributed greatly to the ≈80% reduction in global VL incidence during 2007–2017 (*2*,*4*,*5*).

Local and international health policy makers do not view leishmaniasis as an urgent health issue in Sri Lanka, possibly because of the perceived nonserious nature of CL and relatively small numbers of reported cases (*1*,*2*,*4*). Locally acquired CL was not reported in Sri Lanka before 1992 (*6*), and only a few sporadic cases were reported before incidence rates began to escalate in 2001 (*7*). Since then, locally acquired VL and mucocutaneous forms also have been reported, although most leishmaniasis cases in the country are cutaneous (*7**–**11*). Typical symptoms of CL are single, nontender, nonitchy lesions in the form of nodules, papules, or ulcers (Figure 1, panels A–C) that affect exposed body parts (*7*,*10*). Occasional atypical symptoms include dermal plaques (Figure 1, panel D), erythematous ulcerative patches (*12*,*13*), and mucosal tissue involvement (*14*). In Sri Lanka, initial treatment for CL is weekly intralesional inoculations of sodium stibogluconate administered through dermatology units of the government health sector at a physician’s discretion.

**Figure 1:**
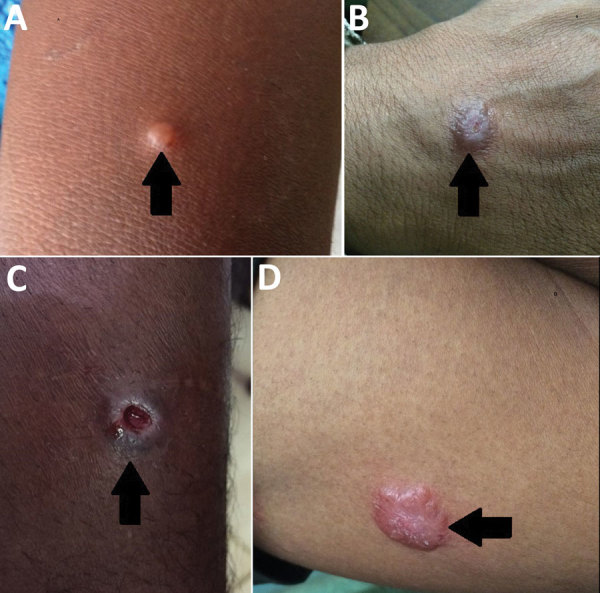
Types of skin lesions observed in cutaneous leishmaniasis case-patients, Sri Lanka, 2001–2018. Arrows indicate A) papule; B) nodule; C) ulcer; D) plaque.

*L. donovani* MON 37, a dermotropic variant of the species, is the causative agent of CL in Sri Lanka but is better known to cause VL elsewhere (*4*,*9*,*15*,*16*). Although the exact basis of dermotropism is unknown, evidence suggests a parasite gene mutation with atypical phenotypic properties manifesting as cutaneous disease devoid of visceralizing features, as noted in long-term patient followup studies (*15**–**17*). The probable vector is the *P. argentipes glaucus* sand fly, which demonstrates zoophilic behavior (*18*,*19*) and differs morphologically and genetically from the *P. argentipes* sensu lato sand fly species found in South India (*20*). 

Local leishmaniasis transmission occurs either outdoors or peridomestically and spatial clustering is seen in highly disease-endemic areas (*10*,*21*,*22*). Despite the global and regional decline in reported leishmaniasis cases, CL incidence has progressed in Sri Lanka since 2001 (*7*,*11*,*22*,*23*). Previous studies on the clinical spectrum, sex and age distribution, spatial clustering of cases, and possible links between climatic and environmental variables led to discussions on challenges to curbing leishmaniasis in the country (*9**–**11*,*22*,*24*). However, no systematic or in-depth studies have been conducted on the epidemiologic characteristics of leishmaniasis in Sri Lanka and its >2-decade progression in the country. Lack of epidemiologic data endangers the health of the population and threatens disease elimination efforts in South Asia, making it a regional, if not global, priority (*3*,*4*,*7*).

The aim of this study was to conduct a retrospective review of the epidemiologic characteristics of leishmaniasis through patient data collected during 2001–2018. The information revealed could inform interventional strategies to address the expansion of leishmaniasis in Sri Lanka, which would improve the likelihood of meeting the goal of South Asia VL elimination plans.

## Methods

We obtained nationwide leishmaniasis data from the diagnostic and research laboratory at the University of Colombo Faculty of Medicine (Colombo, Sri Lanka), which maintains data on laboratory-confirmed CL cases. Laboratory confirmation of CL was made through visualization of *Leishmania* sp. amastigotes upon microscopic examination of Giemsa-stained lesion aspirate smears or slit-skin scrapings (*22*). We also accessed data from the repository of notifiable diseases maintained at the Epidemiology Unit of the Ministry of Health and through communication with medical health officers in small health administrative units in each district. 

Leishmaniasis was made a notifiable disease in Sri Lanka in 2008, at which time notification of cases to the central epidemiology unit became a mandatory requirement. Dermatology units, led by consultant dermatologists, make CL notifications on the basis of strong clinical suspicion with or without laboratory confirmation and have >90% accuracy in local settings (*25*). 

To avoid overlapping patient data, we cross-checked data accessed through different sources. District-level annual CL case counts covered the entire country during 2001–2018. We estimated district- and division-level populations using census data from 2001 and 2012 and projected population levels by assuming a linear annual growth rate. We calculated annual incidence rates for each district as cases per 100,000 population. We mapped case distribution by using ArcGIS 10.1 (Esri, https://www.arcgis.com) and used monthly district-level data from each year to analyze leishmaniasis seasonality.

To analyze disease hotspots, we collected patient data from each division for 2015–2017. We determined hotspots and coldspots by using the Optimized Hot Spot Analysis tool of ArcGIS, which calculates Getis-Ord Gi* spatial statistics (*26*,*27*). We determined hotspots from positive z-scores and coldspots from negative z-scores and CI values of 90%, 95%, and 99% for both. We used Ripley’s K function and the Multi-Distance Spatial Cluster Analysis tool of ArcGIS to determine the average cluster size (*28*). To calculate K function, we used division-level population data from the 2012 Sri Lanka census weighted against leishmaniasis incidence as the clustering variable for each division.

We estimated the population in each district by using census data from 2001 and 2012 with projections for each year as given by the government of Sri Lanka (http://www.statistics.gov.lk). We considered persons from districts with ≥10 cases/100,000 persons/year as at-risk populations.

To analyze age and sex distribution over time and by region, we selected 3 health divisions with the highest incidence rates in the Southern Province, Dickwella, Tangalle, and Beliatta, and 2 health divisions with the highest incidence rates in the North-Central Province, Thalawa and Thamankaduwa. We collected data from 2 periods, 2001–2003 and 2015–2018. We classified age into 3 categories, 0–14, 15–49, and >50 years (*7*,*10*,*29*). We used 2001 and 2012 census data from these divisions for data analysis. We compared differences in sex and age distribution in each region, between regions, and between years by using χ^2^ test. We also compared age distribution against census data by using z-score test with standard residual. We did not analyze sex and age distribution for 2001–2003 because of the low disease incidence.

## Results

### Increasing Trends in Incidence and Spatial Expansion

**Figure 2:**
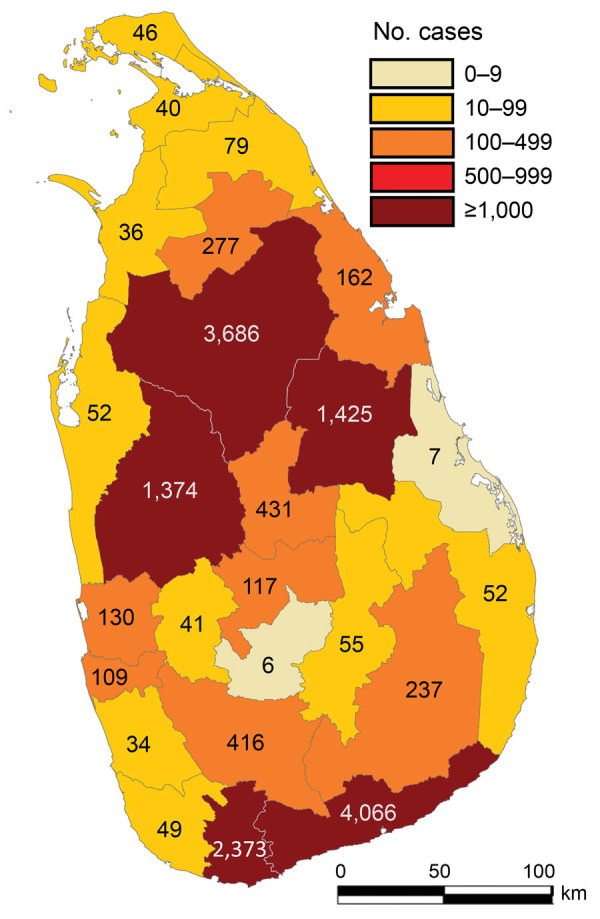
Reported leishmaniasis cases by district, Sri Lanka, 2001–2018.

**Figure 3:**
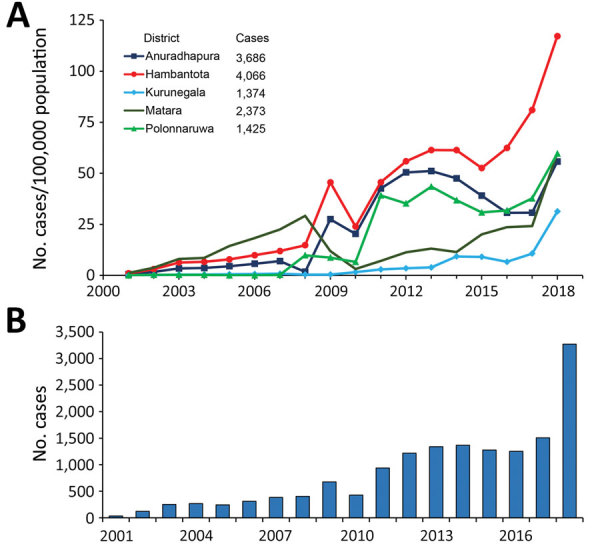
Changes in leishmaniasis incidence and case counts, Sri Lanka, 2001–2018. A) Leishmaniasis incidence rates for 5 districts with the highest numbers of reported cases. B) Nationwide reported leishmaniasis cases by year.

During 2001–2018, island-wide spatial distribution of reported CL cases was 15,300 (Figure 2). Five districts reported >1,000 cases, which accounted for 84.5% (12,924/15,300) of all cases (Figure 2). We noted a slow but steady increase in case numbers from 2001 to 2010, which expanded from 37 to 426 cases/100,000 persons with the majority (73.8%; 2,306/3,125) reported from 3 districts, Hambantota, Anuradhapura, and Matara (Figure 3, panel A). We also noted an increase in incidence during 2010–2011; case counts reached >1,000 during 2012 and remained stable until 2017 (Figure 3, panel B). Most cases (88.3%; 7,865/8,904) were reported from 5 districts, Hambantota, Anuradhapura, Matara, Polonnaruwa, and Kurunegala (Figure 2; Figure 3, panel A). In 2018, we saw an alarming uptick in cases, doubling to 3,271 cases from 1,508 cases in 2017 (Figure 3), and 2 additional districts, Matale and Ratnapura, reached case counts >200 within 1 year. Of the total cases reported during 2018, a total of 86.5% (2,830/3,271) occurred in those 7 districts, and the annual incidence rate was >100 cases/100,000 population in Hambantota District for the first time (Figure 3, panel A). We analyzed monthly data for each year but did not see a uniform seasonal pattern of case distribution at the district, regional, or national level, but we noted that case counts in the north peaked during July–September each year (data not shown).

**Figure 4:**
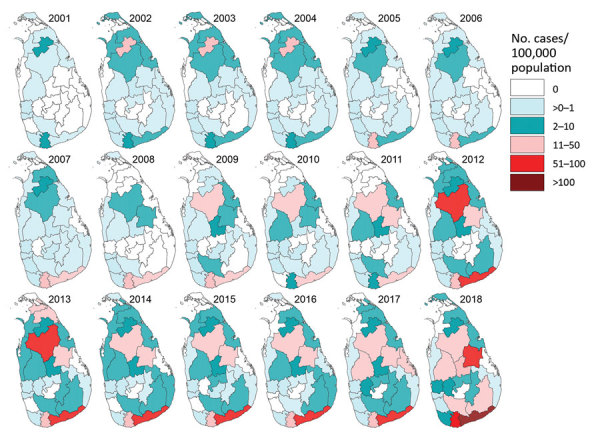
Leishmaniasis incidence rates by district and year, Sri Lanka, 2001–2018.

We saw a marked expansion in spatial distribution of leishmaniasis cases over time (Figure 4). In 2001, the case incidence rate per district was <10 cases/100,000 population, but in 2009, the population living at risk for leishmaniasis increased to >2 million in 3 districts with incidence rates between 10 and 50 cases/100,000 population (Table 1). By 2018, 8 districts had incidence rates of >10 cases/100,000 population, including Hambantota, where the incidence rate reached 117.2 cases/100,000 population (Table 1; Figure 3, panel A). Using 2018 case counts, we estimate 6,622,843 persons, nearly one third of the total population of Sri Lanka, live at considerable risk for leishmaniasis.

**Table 1:** Estimates of population living at risk for leishmaniasis during 2001, 2009, and 2018, Sri Lanka*

Cases/100,000 population	2001			2009			2018	
	No. districts	Population		No. districts	Population		No. districts	Population
0	12	7,728,455		7	3,899,690		2	1,306,933
<1	11	10,157,597		11	11,453,975		6	6,988,158
1–10	2	911,205		4	2,290,870		9	6,622,843
11–50	0	0		3	2,194,176		4	3,862,871
51–100	0	0		0	0		3	2,211,753
>100	0	0		0	0		1	639,340
Total	25	18,797,257		25	19,838,711		25	21,631,898

*Determined by using cutaneous leishmaniasis incidence rates per district and census data from the government of Sri Lanka (http://www.statistics.gov.lk) from 2001 to project potential incidence rates for 2009 and from 2012 to project incidence rates for 2018.

### Shifts in Spatial Distribution and Hotspots 

The increased incidence of leishmaniasis that started in 2001 in 2 districts, Anuradhapura in the North-Central Province and Matara in the Southern Province, extended to other provinces in subsequent years (Figure 4). The disease-endemic area in the North-Central Province expanded during 2001–2018 and its epicenter shifted during 2007–2018 (Figure 4). In the Southern Province, a similar expansion occurred, with a marked increase in incidence rates from 1.2 cases/100,000 persons in 2001 to 117.2 cases/100,000 persons in 2018, but the epicenter remained spatially static (Figure 4).

**Figure 5:**
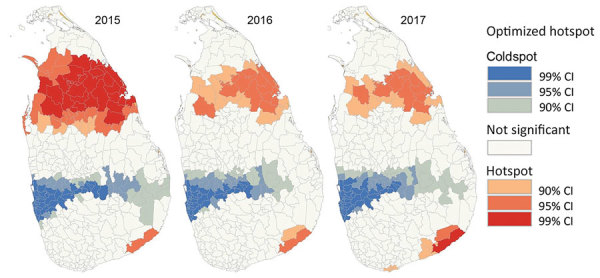
Optimized hotspots and coldspots of leishmaniasis in districts of Sri Lanka during 2015–2017. Hotspots and coldspots were calculated by using the Optimized Hot Spot Analysis tool of ArcGIS (Esri, https://www.arcgis.com); hotspots had large positive z-scores and coldspots had negative z-scores.

**Figure 6:**
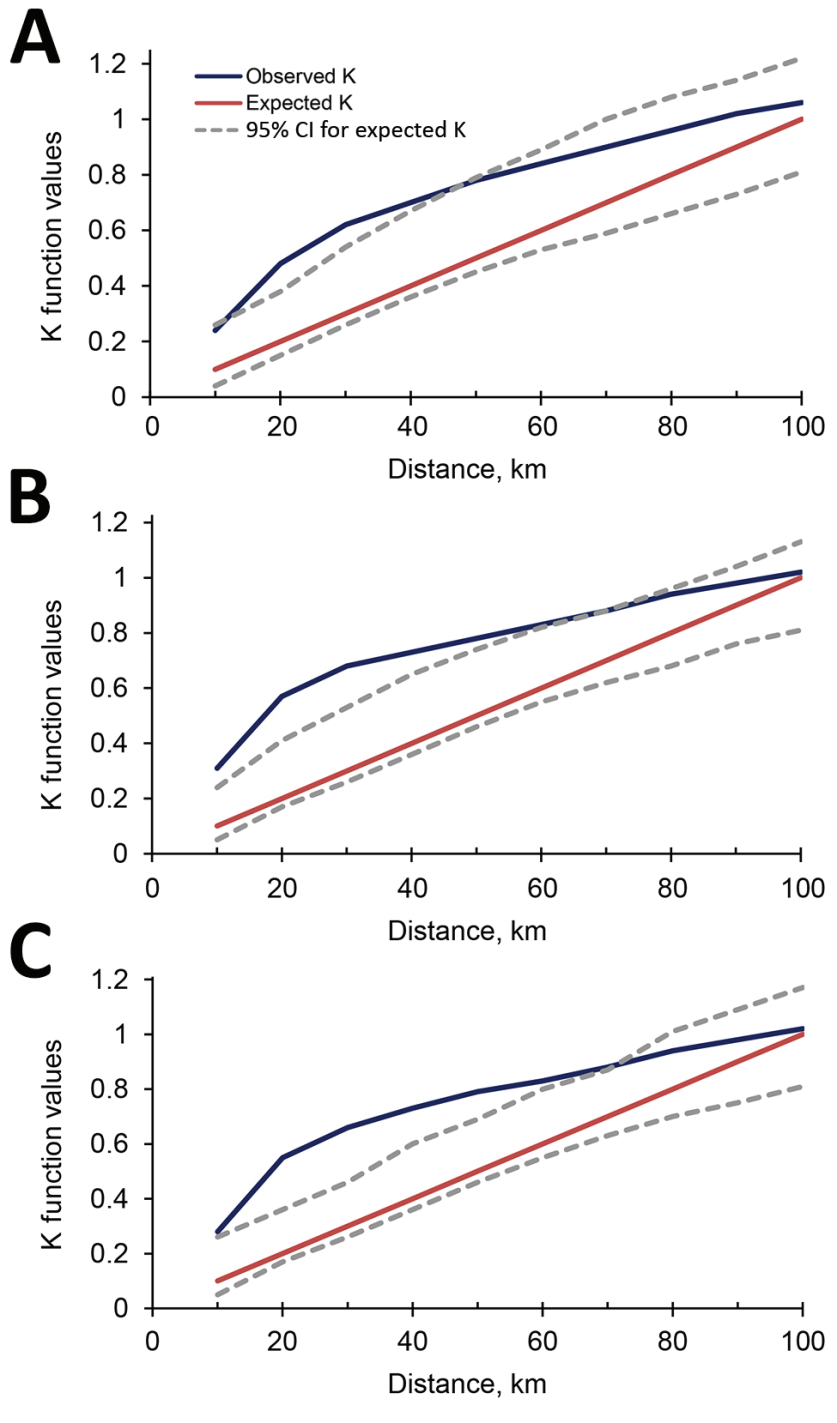
Observed and expected cluster sizes (K values) for incidence of leishmaniasis in Sri Lanka, 2015 (A), 2016 (B), and 2017 (C).

Fine-scale cluster analysis revealed 2 major hotspots in the North-Central and Southern provinces and a coldspot in the central region that spreads across the island from west to east (Figure 5). The size of the hotspot in the North-Central Province gradually shrank, but the one in the southern Province expanded during 2015–2017 (Figure 5). The average size of the Southern hotspot was ≈40 km in radius in 2015 and ≈70 km in 2017 (Figure 6).

### Changes in Age and Sex Distribution over Time

We analyzed anthropometric data of 2,379 cases to study the age and sex distributions and make comparisons between the early disease period, 2001–2003, and the late period, 2015–2018, and between the North-Central and Southern provinces (Table 2). We noted 200 cases during 2001–2003 and 2,179 cases during 2015–2018 and a statistically significant change in the age and sex distribution in the North-Central Province between the early and late periods (Table 2). During 2001–2003, most cases (94.7%) in the North-Central Province were in male patients, but the proportion of male cases dropped to 68.8% during 2015–2018 (χ^2^ = 38.75, df = 1, p<0.0001). Similarly, in this region most cases (86.3%) were in persons 15–49 years of age during the early period but dropped to 53.7% in this group during the late period (χ^2^ = 47.73, df = 1, p<0.0001) (Table 2). In contrast, in the Southern Province the sex distribution remained the same in the early and late periods (χ^2^ = 0.0001, df = 1, p = 0.9784) (Table 2), but the age range of the highest incidence group shifted from persons >50 years of age (40.6%) during the early period to those 15–49 years of age (54.7%) during the late period (χ^2^ = 10.62, df = 2, p = 0.0049) (Table 2).

**Table 2:** Characteristics of leishmaniasis cases in selected highly disease-endemic areas of 2 provinces, Sri Lanka*

Characteristics		North-Central Province			Southern Province	
		2001–2003	2015–2018		2001–2003	2015–2018
Age, y						
0–14		6 (4.6)	70 (11.3)		17 (24.6)	256 (16.4)
15–49		113 (86.3)	332 (53.7)		24 (34.8)	854 (54.7)
≥50		12 (9.2)	216 (35.0)		28 (40.6)	451 (28.9)
Sex						
F		7 (5.3)	198 (32.0)		28 (40.6)	636 (40.7)
M		124 (94.7)	420 (68.0)		41 (59.4)	925 (59.3)
Total		131	618		69	1,561

*Values represent no. (%) in each age and sex category during early and late periods of disease expansion. Data provided by medical health officers in each region.

Further analysis of data from the late period revealed statistically significant differences in distribution of the disease by sex within and between the North-Central and Southern provinces (Table 3). Further examination revealed fewer cases in persons <14 years of age, but more male patients in this age group, a finding common to both regions (Table 3). Regardless of sex, we noted a higher incidence rate in those >50 years of age in the North-Central Province compared with census data, but we did not see this pattern in the south (Table 3). In addition, we did not see a difference in age distribution of female patients between the 2 regions (χ^2^ = 0.69, df = 2, p = 0.7065), but we saw a statistically significant difference in male patients (χ^2^ = 27.74, df = 2, p<0.0001) (Table 3) relative to the age distribution reported in census data. Our findings demonstrate a statistically high incidence rate in persons >50 in the North-Central Province compared with the Southern Province.

**Table 3:** Differences in sex and age distribution in cases of leishmaniasis against census data for regions of Sri Lanka, 2015–2018*

Characteristics by province	F, no. (%)	M, no. (%)	Total		χ^2^	df	p value
North-Central	198 (32.0)	420 (68.0)	618		159.50	1	<0.001
Southern	636 (40.7)	925 (59.3)	1,561		107.01	1	<0.001
North-Central versus Southern					14.20	1	0.002
			Standard residuals†				
Age ranges by province, y			F	M			
North-Central							
0–14	39 (19.7)	31 (7.4)	−1.29	**–3.85**	20.38	2	<0.001
15–49	95 (48.0)	237 (56.4)	−0.75	0.56			
≥50	64 (32.3)	152 (36.2)	**2.70**	**3.62**			
Southern							
0–14	49 (25.5)	37 (13.9)	−0.96	**–2.27**	37.76	2	<0.001
15–49	76 (39.6)	162 (60.7)	−0.49	1.60			
≥50	67 (34.9)	68 (25.5)	1.55	0.07			
North-Central versus Southern							
F by age group					0.69	2	0.7065
M by age group					27.74	2	<0.001

*Leishmaniasis case count data provided by regional medical health officers. Where not specified, comparisons were made between the number of female and male cases in different age groups within the same region. df, degrees of freedom. †Age distributions by sex standardized against census data from the same regions. Numbers in bold indicate statistically significant difference from census (p<0.05).

## Discussion

Leishmaniasis was seldom reported in Sri Lanka before the 1990s (*30*), and neither local nor international health authorities have considered it a serious public health threat in the country (*1*,*2*,*4*). However, as the case incidence and spread intensifies, leishmaniasis is increasingly becoming a concern, especially among residents of Sri Lanka (*7*,*11*,*24*,*31*). Furthermore, 2 major disease hotspots emerged during 2001–2003 and disease-affected areas expanded during 2011–2018, reaching >3,000 cases nationwide in 2018, a drastic increase from preceding years. The actual picture might be worse because reports from health facilities reflect only a fraction of the true incidence (*7*,*23*,*31*,*32*). Many questions regarding leishmaniasis in Sri Lanka remain unanswered and no organized efforts are in place for its control at a national level, or even in disease hotspots.

The alarming case expansion in 2018 could reflect a buildup of asymptomatic or early-stage symptomatic infections in the preceding years, but no field data are available to support this hypothesis. The infection-reservoir pool also might have grown because of poor treatment response, a growing problem in local healthcare settings (*13*). The 2001 increase in cases began in an army camp adjoining a jungle in the North-Central Province during a civil war (*7*)*.* The initial cases could be attributed to known risk factors, such as nonimmune hosts entering the vector’s habitat (*33*,*34*). Although it was suspected at the outset, no zoonotic reservoir has been proven to be the cause of the upsurge in cases, but leishmaniasis later was detected in dogs in Sri Lanka (*35*,*36*). Increased population mobility in the country after the civil war ended in 2009, along with enhanced infrastructure developments and easy road access, could have facilitated the spread of leishmaniasis. Activities raising CL awareness among the population also might have increased self-referrals and improved case diagnoses, thus contributing to progressive increases in case numbers. However, none of these factors, taken singly or in combination, can explain the case upsurge observed in 2018, highlighting the need for in-depth longitudinal studies. 

Genetic analysis through microsatellite typing and whole-genome sequencing suggests prolonged existence of *Leishmania* spp. in the country and refutes theories of recent parasite introduction (*15*,*37*). The endemic *Leishmania* parasitic population could have been expanding gradually and spreading within foci, forming focal clusters (*22*). We hypothesize that during this phase of parasitic population growth, asymptomatic disease reservoirs were created and later expanded, contributing to the sudden increase of case numbers in 2018. However, carefully designed cross-sectional field studies are required to confirm this hypothesis. 

Malaria cases have declined in Sri Lanka since 2000, and the last indigenous case was reported in 2012 (*38*). Subsequent restrictions on insecticide use for vector control could have played a role in the increased incidence of leishmaniasis in the intervening years. Leishmaniasis control is a widely recognized byproduct of concerted malaria control in the region (*39*). Reduced vector control for malaria could explain the contrasting pictures, almost mirror images, of declining incidence rates for malaria and increasing rates of leishmaniasis in Sri Lanka during the past 2 decades.

Reporting on patients with leishmaniasis has improved over the years, as has the level of disease awareness among clinicians and healthcare personnel. These factors could have contributed to the increased case documentation, but underdiagnosis remains a concern (*11*,*23*,*32*). Local travel and vector dispersal also are factors that cannot be ruled out and might have contributed to the 2018 case surge, since these expansions were adjacent to the previous areas of disease (*23*,*40*,*41*).

Although leishmaniasis affects both sexes in all age groups, previous studies consistently indicated a male predominance among cases in groups 20–40 years of age (*7*,*12*,*23*,*31*,*32*). Our study showed differences in the sex and age distribution between the northern and southern disease foci. In southern regions, the incidence data deviated from the census data with reduced numbers of disease in patients <14 years of age, and more so in male patients. Other age groups were equally affected by the disease, even in the early period, except this age group, raising concerns of under reporting and undiagnosed cases among children, especially boys, and creating a potential for them to become disease reservoirs. The predominance of young men (20–40 years) infected during the early period easily could be explained by the disease foci located in a military camp in the North-Central Province (*7*,*10*). However, for 2015–2018, persons >50 years of age in both sexes had much higher incidence rates compared with other age groups, out of proportion with trends seen in census data. One or more factors, such as changes in the level of infection awareness, behavioral differences, environmental factors, vector-related factors, and peridomestic transmission patterns, might have contributed to such findings and warrant further investigation (*10*,*22*,*42*). Enhanced surveillance is needed to ensure coverage of all age groups, including children.

We noted a clear expansion of spatial distribution in reported CL cases. Because *L. donovani* also is the causative agent for VL, the expansion of CL in Sri Lanka could be a potential threat to the regional VL elimination efforts (*2*). The VL elimination target is <10 cases/100,000 population/year, an incidence rate at which the disease is no longer considered a public health concern (*2*). If similar standards had been applied to *L. donovani*–induced CL in Sri Lanka, leishmaniasis would not have been public health concern in 2001 and might have been considered a minor concern until 2009, when only 1 district had >10 cases/100,000 population. However, under this target, leishmaniasis in Sri Lanka should be considered a major public health threat, especially considering our calculations show that more than one third of country’s population is at risk for this infection. 

We did not see the clear-cut pattern of seasonality described in previous studies, which demonstrated cases increased in a district in 2 biannual peaks (*43*). However, peak case numbers in the north during July–September might be related to seasonal vector abundance, which needs confirmation. The hotspots detected in the north and the south are likely caused by the expansion of local disease transmission. Although the epicenters of disease shifted over time, they remained in the same broader areas where they started in 2001. The central highlands appear to act as a barrier for disease spread, probably because environmental factors do not favor the survival of the vector sand flies.

Transmission of leishmaniasis in Sri Lanka is likely to progress, unless active interventions for disease containment are put in place. In the absence of proof for the presence of nonhuman reservoirs, infection control measures should focus on early diagnosis and effective treatment for patients; vector control involving chemical and environmental methods; and reducing human–vector contact by educating the population on steps they can take to reduce their risk for infection, such as applying insect repellents and using insecticide-impregnated bed nets. However, specific vector control measures essentially require more studies on vector behavior and insecticide susceptibility to inform evidence-based policy decisions. 

Carefully designed longitudinal studies are needed in the community to clarify the epidemiology and transmission dynamics of the disease. Intensive awareness programs should be implemented for clinicians and healthcare workers to ensure effective patient management, and for the general public to improve treatment-seeking behavior, backed up by qualitative studies to enhance early case detection. Better accessibility and the use of more cost-effective treatment options, such as radiofrequency heat therapy (*44*), could improve patient compliance and reduce infection reservoirs. Furthermore, use of modern technological tools, such as satellite remote sensing, could aid in epidemiologic surveillance, identification of probable sandfly-infested areas, and prediction of disease hotspots. In addition, planning and implementation of effective interventions would improve containment efforts for leishmaniasis in Sri Lanka.
